# Supplementary material for: Graphdiyne oxide nanosheets display selective anti-leukemia efficacy against DNMT3A-mutant AML cells
Source: Nat Commun. 2022 Sep 26;13:5657. doi: 10.1038/s41467-022-33410-w (PMC9512932; doi:10.1038/s41467-022-33410-w)
Supplement: Supplementary file 1 — Supplementary Information [file 41467_2022_33410_MOESM1_ESM.pdf]

Supplementary Information

Graphdiyne oxide nanosheets display selective anti-leukemia efficacy against DNMT3A-mutant AML cells

Qiwei Wang. *et al*

Corresponding authors:

*Pengxu Qian

Investigator

School of Medicine, Zhejiang University

Email: axu@zju.edu.cn

* Chunying Chen

Investigator

National Center for Nanoscience and Technology of China

Email: chenchy@nanoctr.cn

The PDF file includes:

Supplementary Figure 1 to 40

Other Supplementary Material for this manuscript includes the following:

Supplementary Movies 1 to 6

Supplementary Data 1 to 4

Supplementary Figures

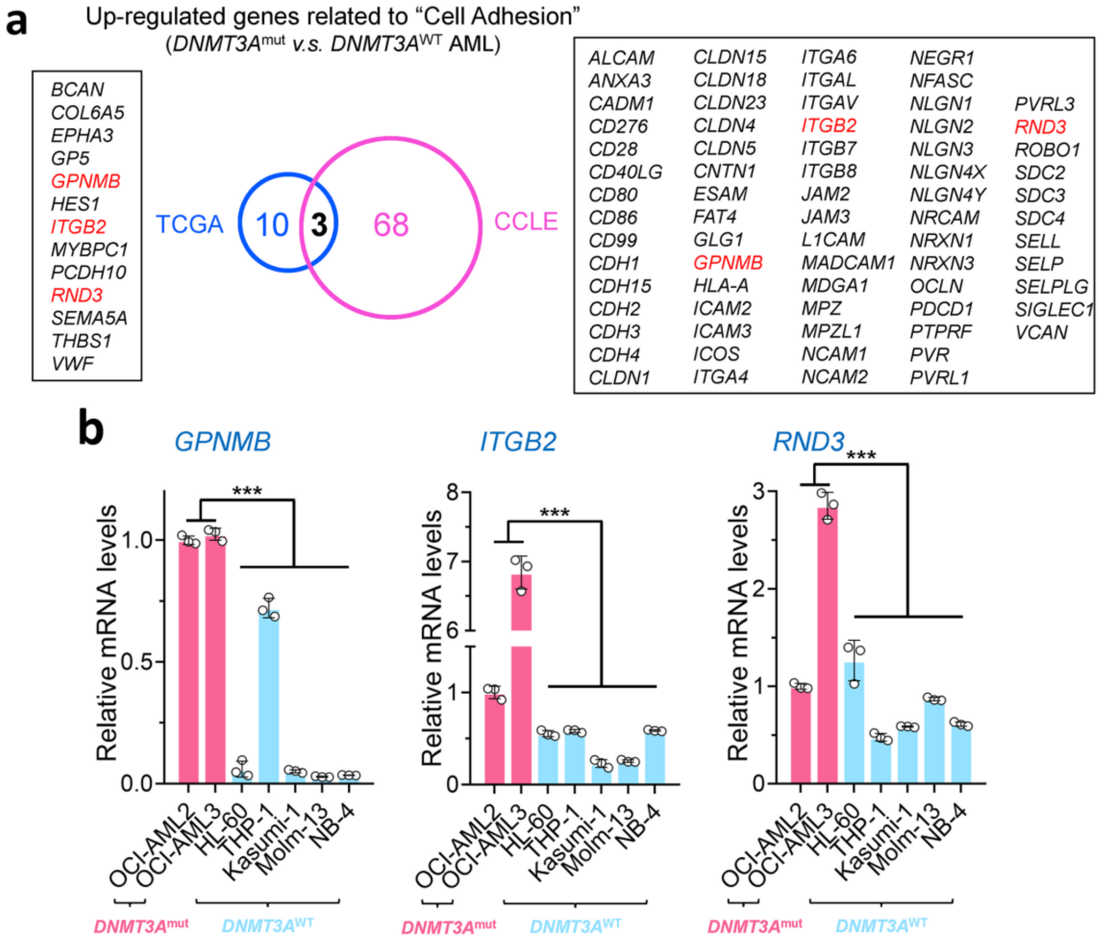

Supplementary Figure 1. Up-regulated genes related to "Cell Adhesion" in *DNMT3A*-mutant AML. a, Venn diagram of up-regulated genes in *DNMT3A*-mutant AML cells isolated from both AML patients (TCGA: *DNMT3A*^{mut}, n = 36; *DNMT3A*^{WT}, n = 115) and AML cell lines (CCLE: *DNMT3A*^{mut}, n = 4; *DNMT3A*^{WT}, n = 30). **b,** Q-PCR verifications of three overlapped genes of TCGA and CCLE databases in human AML cell lines. n = 3 biologically independent experiments. The data were shown as the mean ± SD. Statistical significance was tested with One-way ANOVA test. ***$P < 0.001$.

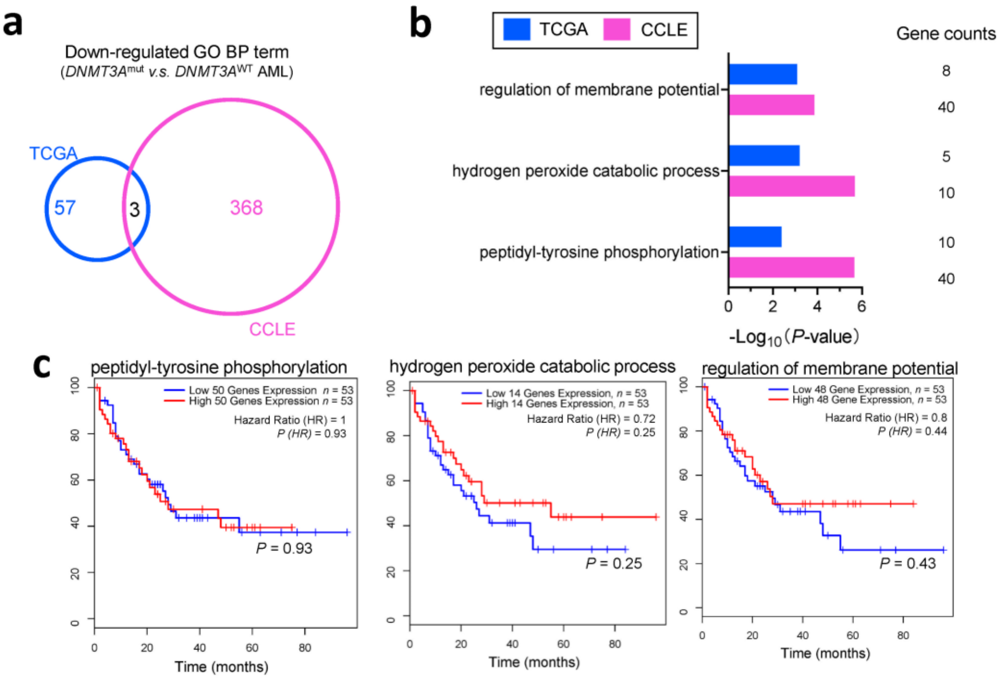

Supplementary Figure 2. Down-regulated biological process (BP) terms in *DNMT3A*-mutant AML. a, Venn diagram of down-regulated BP terms in DNMT3A-mutant AML cells isolated from both AML patients (TCGA: *DNMT3A*^{mut}, n = 36; *DNMT3A*^{WT}, n = 115) and AML cell lines (CCLE: *DNMT3A*^{mut}, n = 4; *DNMT3A*^{WT}, n = 30). **b,** Three down-regulated BP terms in the intersection and corresponding gene counts. The statistical tests were one-sided and adjustments were made for multiple comparisons. **c,** Survival curves of AML patients with high and low expression designated gene sets, and n is the number of biologically independent samples. The survival analyses were performed by the log-rank (Mantel–Cox) test.

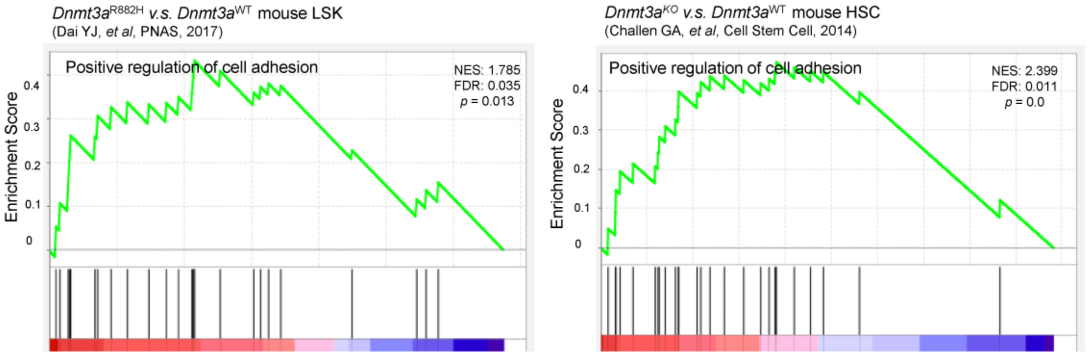

Supplementary Figure 3. Gene set enrichment analysis (GSEA) of "Positive regulation of cell adhesion" in *Dnmt3a*^{R882H} mouse LSK cells and *Dnmt3a*$^{-/-}$ mouse HSCs. The statistical tests were one-sided and adjustments were made for multiple comparisons.

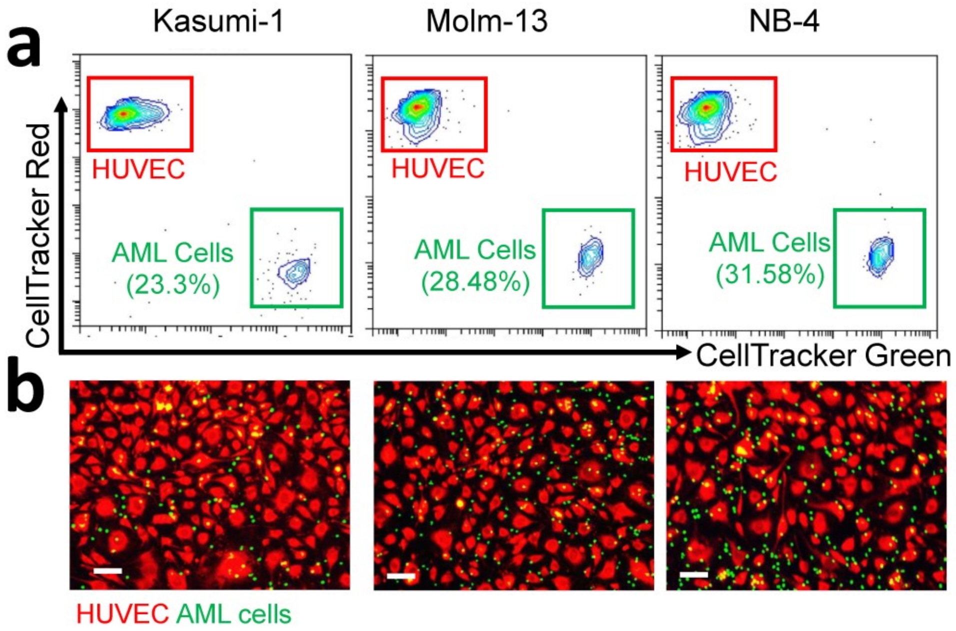

Supplementary Figure 4. Cell-cell adhesion between AML cells and HUVECs. Flow cytometry data **(a)** and fluorescent images **(b)**, Scale bar, 50 μm.

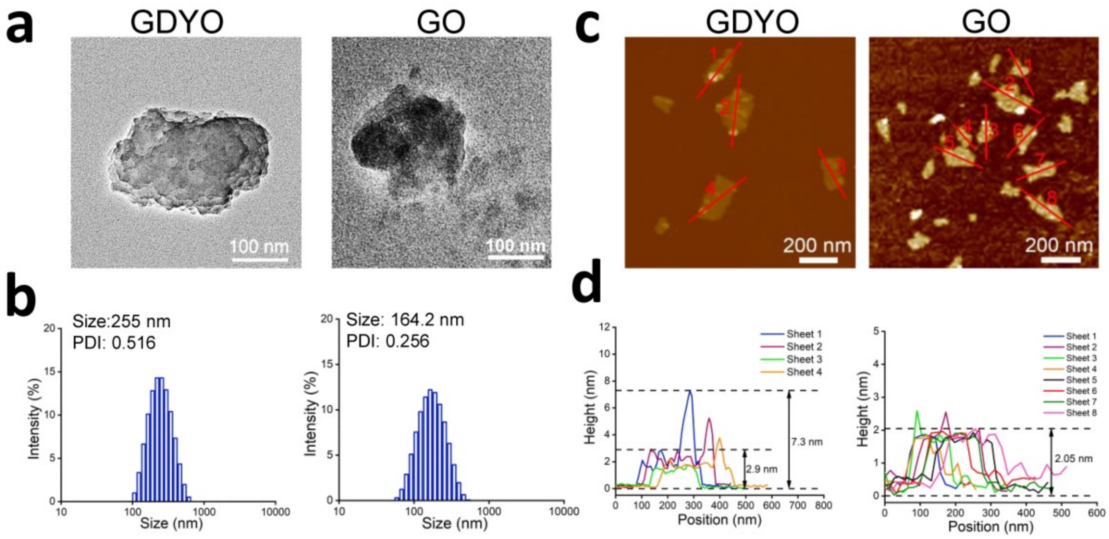

Supplementary Figure 5. Characterization of GO and GDYO nanosheets. a-b, TEM and DLS (Dynamic light scattering) characterization for GDYO and GO. Scale bar, 100 nm. **c-d,** AFM (Atom force microscope) imaging (c) and (d) height characterization for GDYO and GO. Scale bar, 200 nm. **a** and **c,** at least 20 images for each sample were taken.

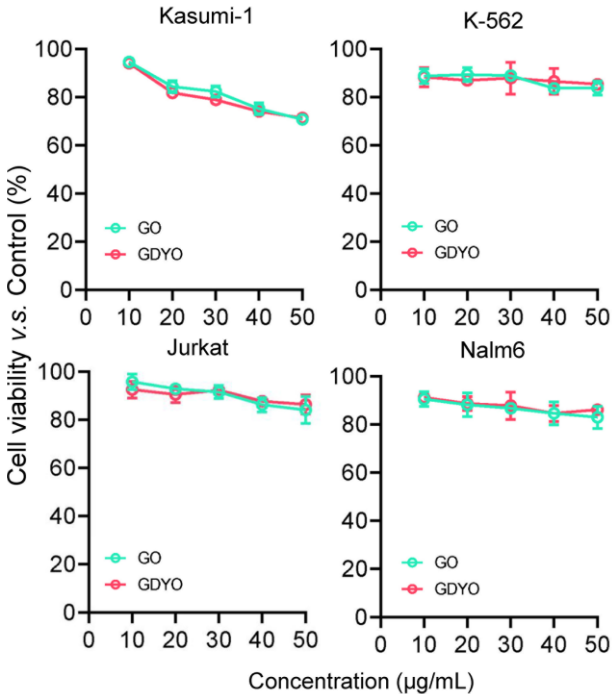

Supplementary Figure 6. Cell viability assay of DNMT3A wildtype leukemia cell lines (Kasumi-1, K-562, Jurkat and Nalm6) treated with GO or GDYO at different concentrations for 24 h. *n* = 3 biologically independent experiments. The data were shown as the mean ± SD. Statistical significance was tested with a two-tailed, unpaired Student's *t* test.

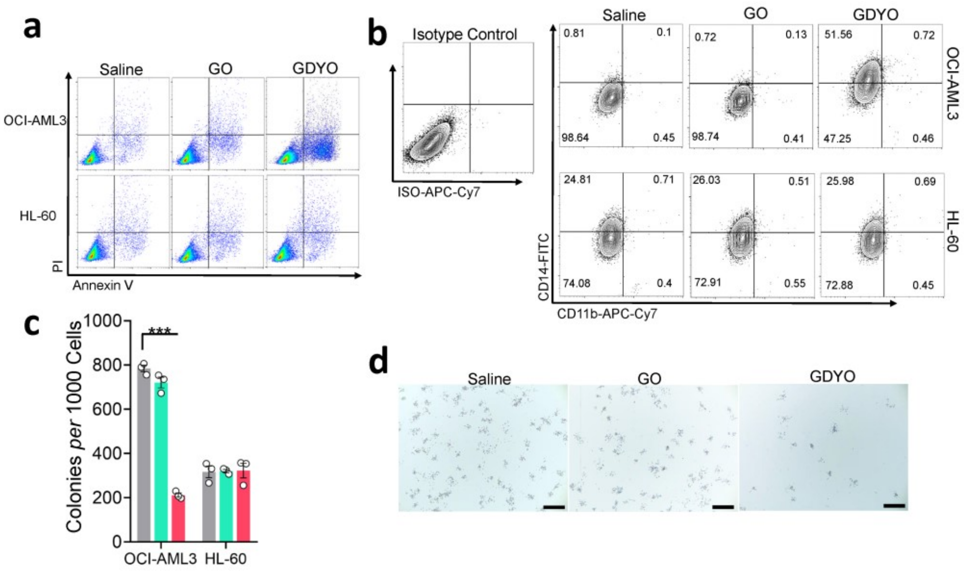

Supplementary Figure 7. Representative images for apoptosis, myeloid differentiation and CFU in cells treated with GO or GDYO. a-b, Representative flow cytometry images for cell apoptosis **(a)** and myeloid differentiation **(b)**. **c,** CFU colonies formation of 1000 cells after 10 d culture in methylcellulose medium. *n* = 3 biologically independent experiments. The data were shown as the mean ± SD. Statistical significance was tested with a two-tailed, unpaired Student's *t* test. ****P*< 0.001. **d,** CFU colonies images after 10 d culture in methylcellulose medium for OCI-AML3. At least 10 images for each sample were taken. Scale bar, 200 μm.

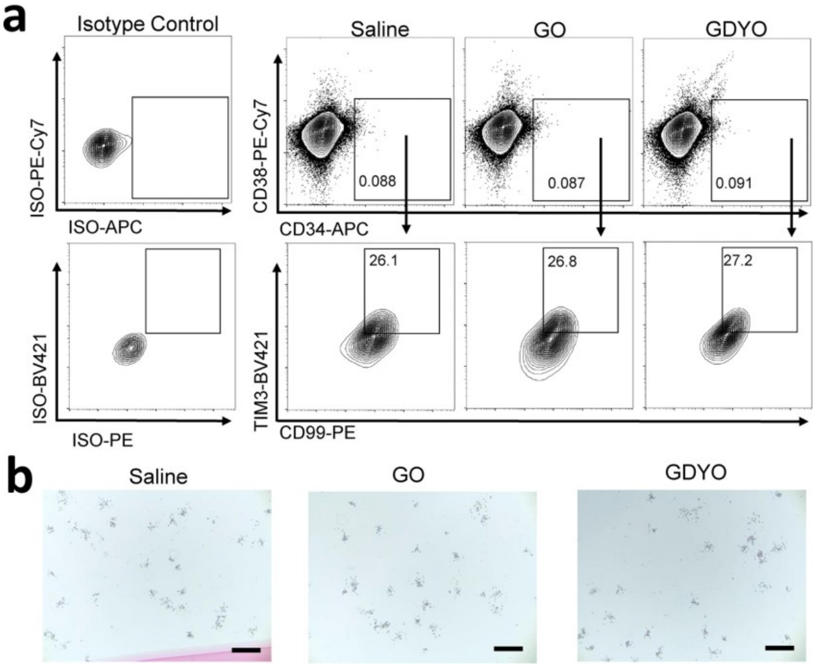

Supplementary Figure 8. Representative flow cytometry images for leukemia stem cells (a) and microscopy images of CFU (b) in HL-60 treated with GO or GDYO. b, At least 10 images for each sample were taken. Scale bar, 200 μm.

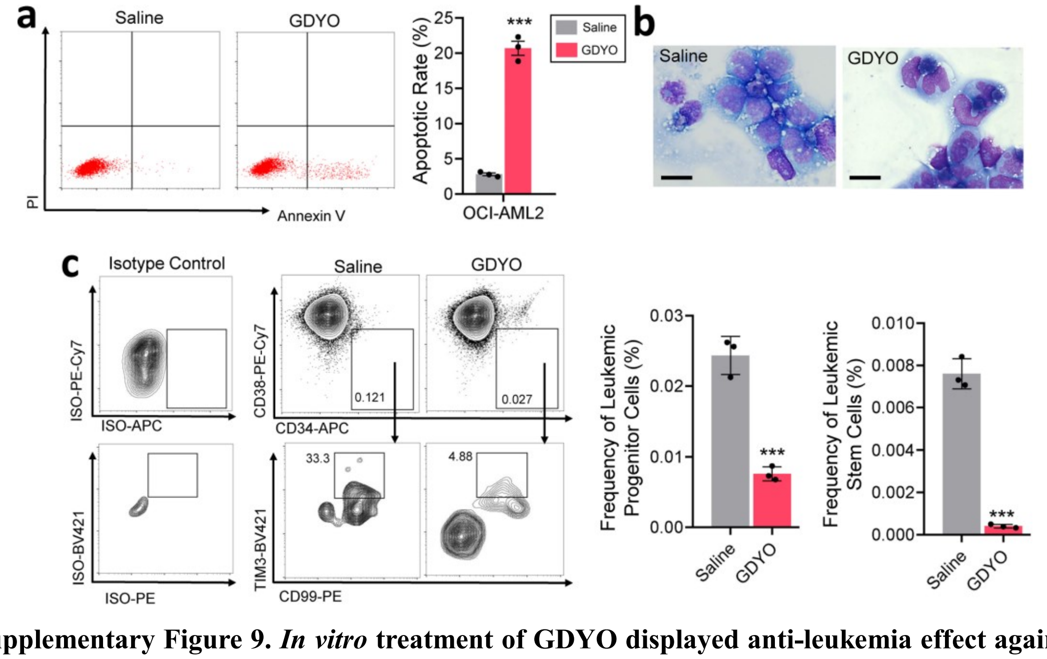

Supplementary Figure 9. *In vitro* treatment of GDYO displayed anti-leukemia effect against *DNMT3A* mutant AML cells (OCI-AML2). a, Apoptosis analysis in OCI-AML2 treated with 20 μg/mL GO/GDYO for 48 h. $n = 3$ biologically independent experiments. **b,** Wright-Giemsa staining showing signs of maturation in GDYO-treated OCI-AML2. Scale bar, 10 μm. **c.** Analysis of leukemic progenitor cells and leukemic stem cells in OCI-AML2 treated with 20 μg/mL GO/GDYO for 72 h. $n = 3$ biologically independent experiments. The data were shown as the mean ± SD. Statistical significance was tested with a two-tailed, unpaired Student's t test. ***$P < 0.001$.

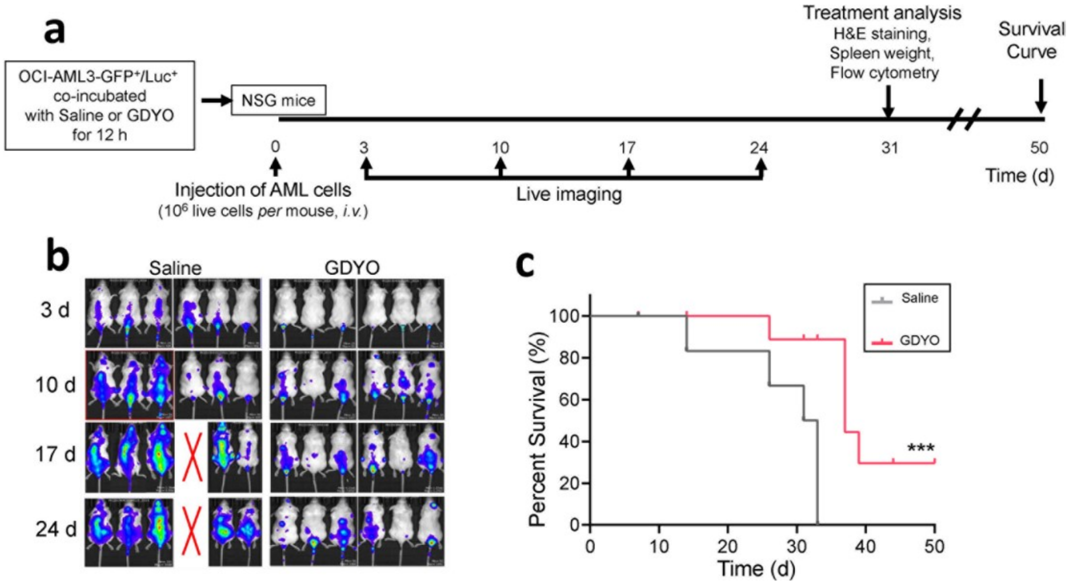

Supplementary Figure 10. *In vitro* GDYO treatment inhibited *in vivo* leukemogenesis of *DNMT3A*-mutant AML cells. a. Schematic illustration of the experimental design. **b.** Representative IVIS spectrum images of AML NSG mice, $n = 6$ biologically independent mice. **c.** Kaplan-Meier survival curves of AML mice injected with saline or GDYO. The survival analysis was performed by the log-rank (Mantel–Cox) test. $^{***}P < 0.001$.

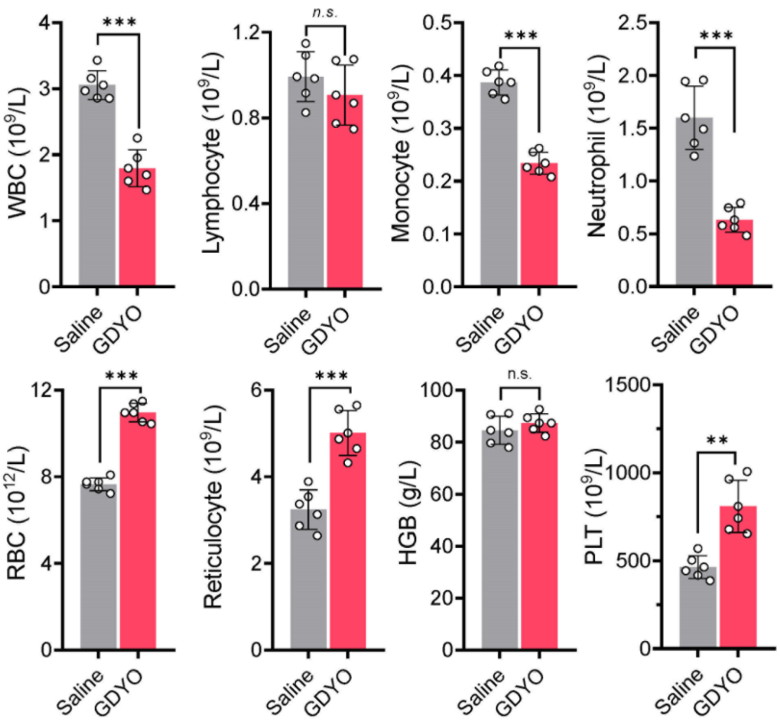

Supplementary Figure 11. Hematological data of AML mice. The AML cells were pre-treated with saline or GDYO. WBC, white blood cell, RBC, red blood cell, HGB, hemoglobin, PLT, platelets. $n = 6$ biologically independent animals. The data are shown as the mean ± SD. Statistical significance was tested with a two-tailed, unpaired Student's *t* test. *n.s.* no significant, $^{**}P < 0.01$, $^{***}P < 0.001$.

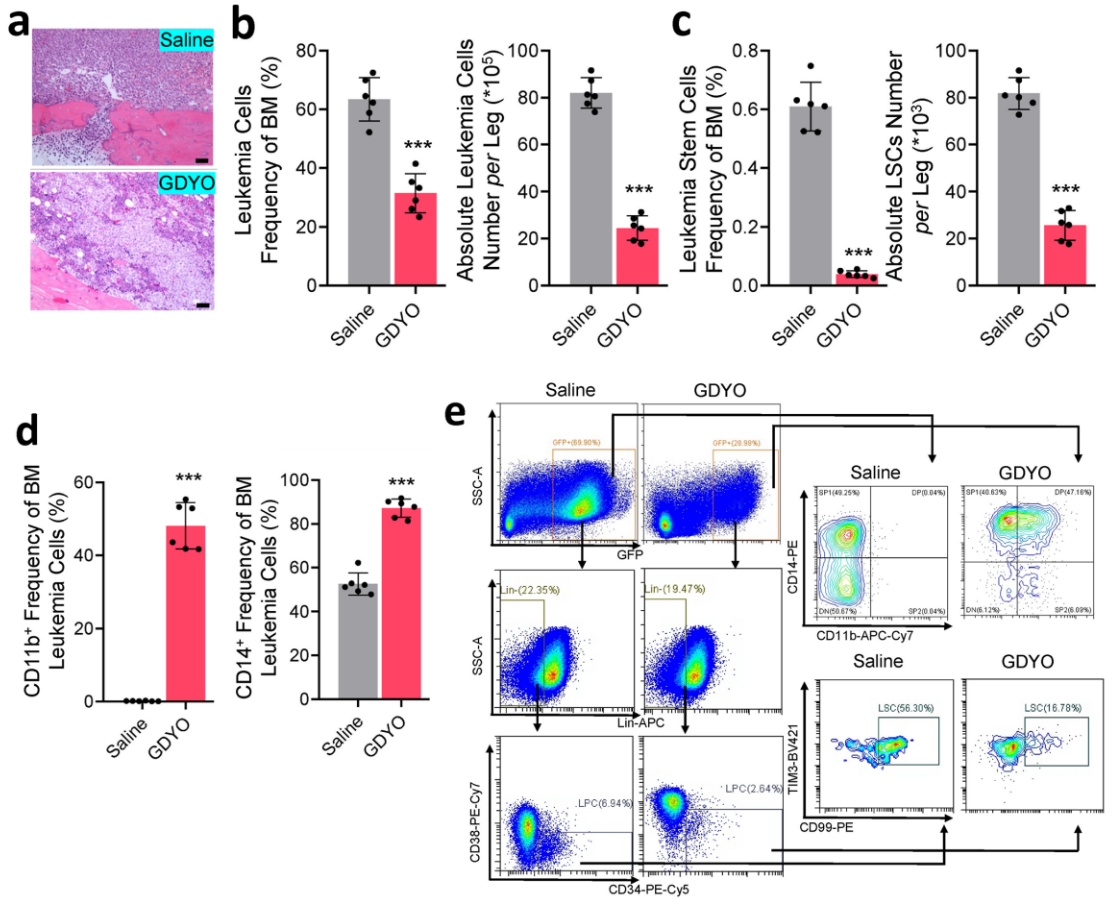

Supplementary Figure 12. Bone marrow (BM) analysis of AML mice. The AML cells were pre-treated with saline or GDYO. **a,** H&E staining on bone marrow sections of AML mice injected with saline or GDYO. Scale bar, 10 μm. **b-d,** Quantification of leukemia cells **(b)**, leukemic stem cells **(c)** and myeloid differentiation **(d)** in bone marrow. **e,** Representative flow cytometry analysis of bone marrow total mononuclear cells. $n = 6$ biologically independent animals. The data were shown as the mean ± SD. Statistical significance was tested with a two-tailed, unpaired Student's t test. ***$P < 0.001$.

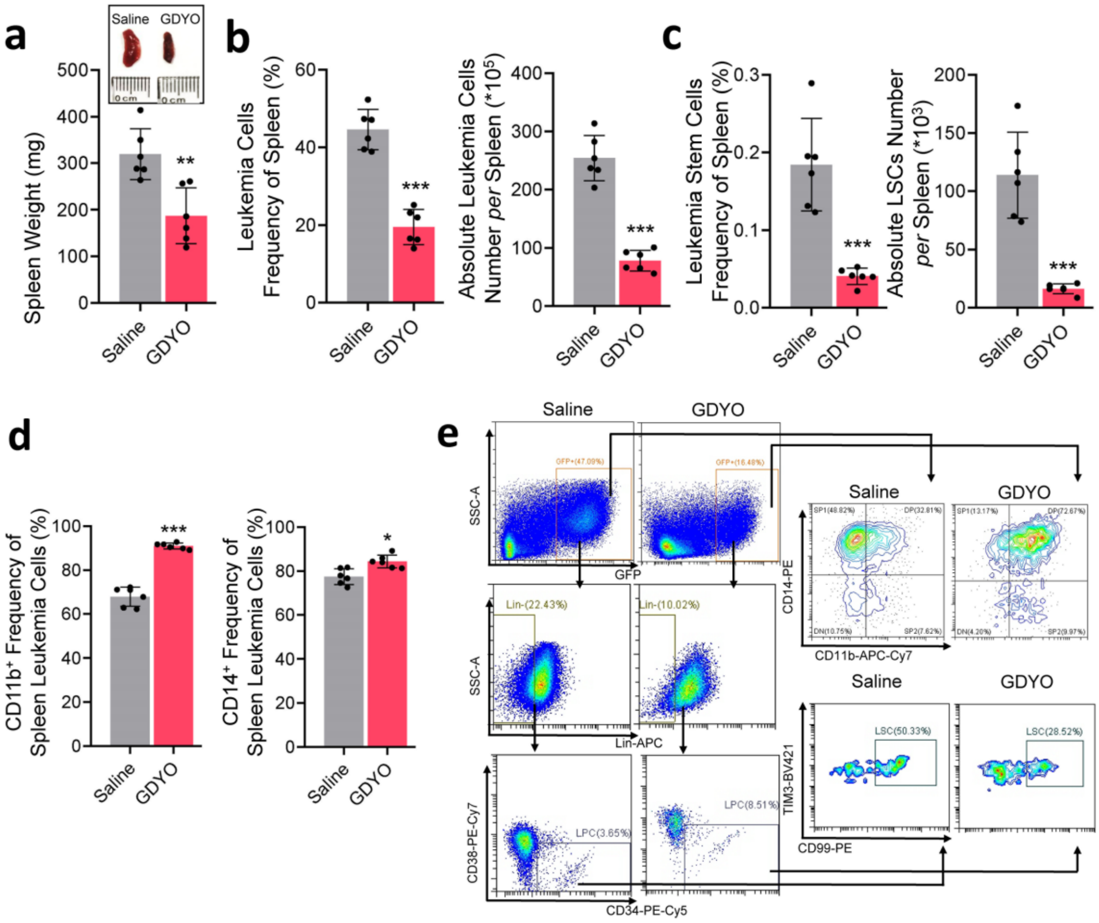

Supplementary Figure 13. Spleen analysis of AML mice. The AML cells were pre-treated with saline or GDYO. **a,** Spleen weights of AML mice injected with saline or GDYO at sacrifice. Scale bar, 10 μm. **b-d,** Quantification of spleen leukemia cells **(b)**, leukemic stem cells **(c)** and myeloid differentiation **(d)**. **e,** Representative flow cytometry analysis of spleen total mononuclear cells. $n = 6$ biologically independent animals. The data were shown as the mean ± SD. Statistical significance was tested with a two-tailed, unpaired Student's *t* test. *$P < 0.05$, **$P < 0.01$, ***$P < 0.001$.

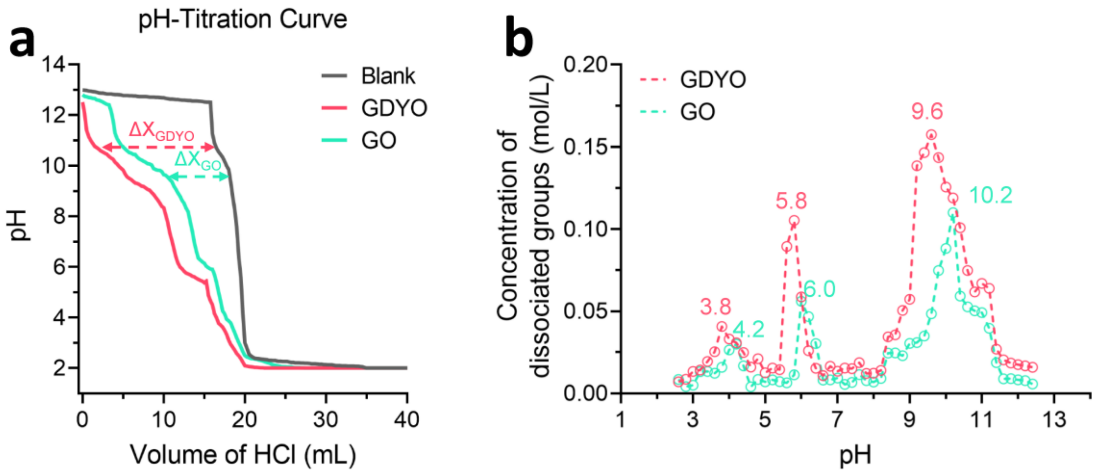

Supplementary Figure 14. pH titration and acid dissociation curves of GDYO and GO. (a) The pH titration curves of GDYO and GO. Δ indicated the different volume of HCl added in the two titrations at the same value of pH, given the concentrations of the ionized groups *per* g of GDYO or GO at that pH. **(b)** The experimental (circle) and simulated (dotted line) acid dissociation curves of GDYO and GO dispersions.

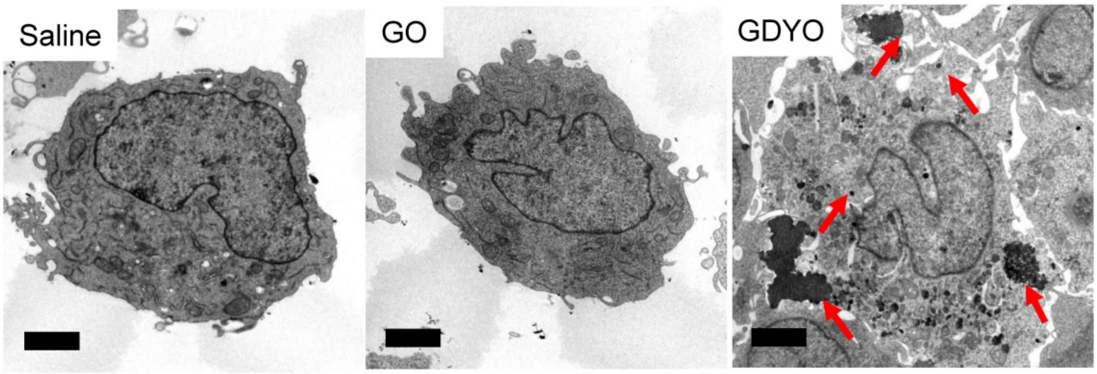

Supplementary Figure 15. Representative TEM images for OCI-AML3 treated with Saline/GO/GDYO for 48 h. At least 15 images for each sample were taken. Scale bar, 2 μm.

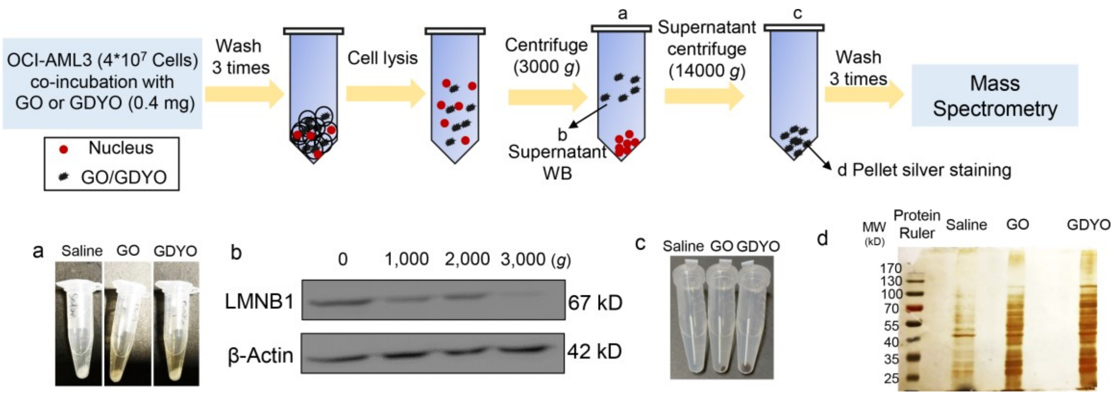

Supplementary Figure 16. Sample preparation diagram of pull-down protocol to analyze GDYO-interacted proteins. a, c, Photos of the corresponding steps. **b,** 3,000 *g* maximizes nuclear removal while reducing GDYO loss. **d,** Silver staining of each samples for LS-MS. n = 2 biologically independent experiments.

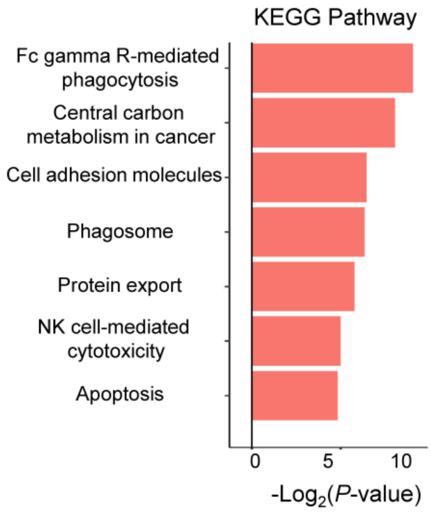

Supplementary Figure 17. KEGG pathway of GDYO-interacted proteins. The statistical tests were one-sided and adjustments were made for multiple comparisons.

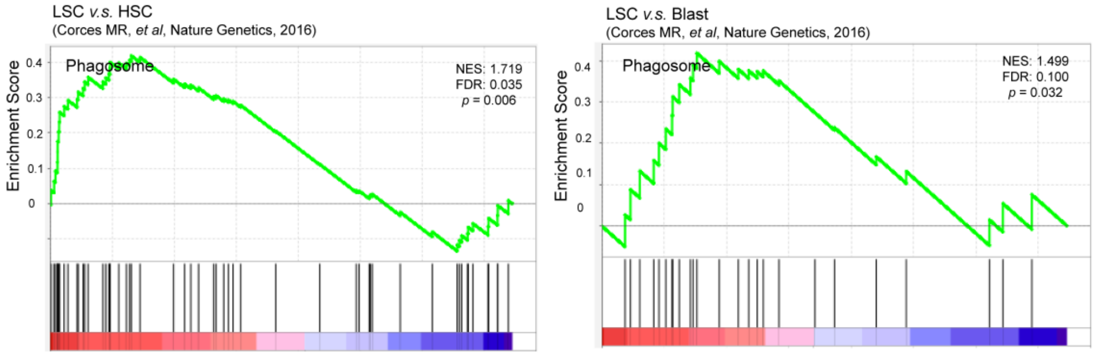

Supplementary Figure 18. Gene set enrichment analysis (GSEA) in LSC *v.s.* HSC and LSC *v.s.* Blast cells for "phagosome". The statistical tests were one-sided and adjustments were made for multiple comparisons.

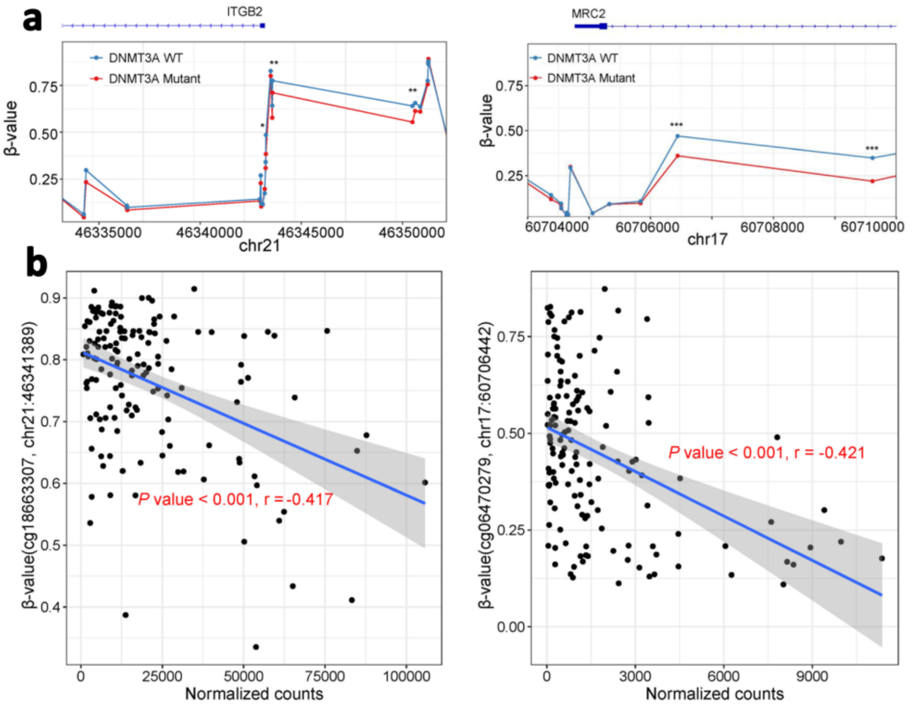

Supplementary Figure 19. Methylation analysis of ITGB2 and MRC2 in *DNMT3A*-mutated AML. **a**, Differentially methylated regions (DMRs) for ITGB2 and MRC2 in *DNMT3A* mutant and *DNMT3A* wildtype AML isolated from TCGA. *DNMT3A*-mutant, $n = 49$; *DNMT3A*-wt, $n = 145$. The statistical tests were one-sided and adjustments were made for multiple comparisons. $*p < 0.05$, $**p < 0.01$, $***p < 0.001$.**b**, Correlation analysis between DMRs and gene expression for ITGB2 and MRC2 in *DNMT3A* mutant and *DNMT3A* wildtype AML isolated from TCGA. *DNMT3A*-mutant, $n = 49$; *DNMT3A*-wt, $n = 145$. The statistical tests were one-sided and adjustments were made for multiple comparisons. The blue lines represented the fitted curve and the shaded area as 95% confidence interval.

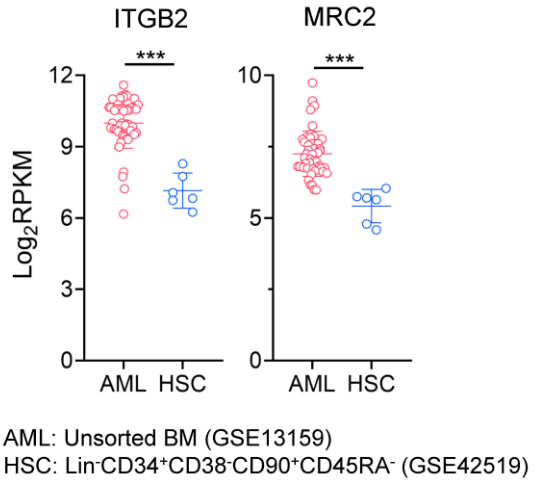

Supplementary Figure 20. Expression of ITGB2 and MRC2 in human AML cells and HSCs. AML, $n = 48$; HSC, $n = 6$. The data were shown as the mean ± SD. Statistical significance was tested with a two-tailed, unpaired Student's t test. $***p < 0.001$.

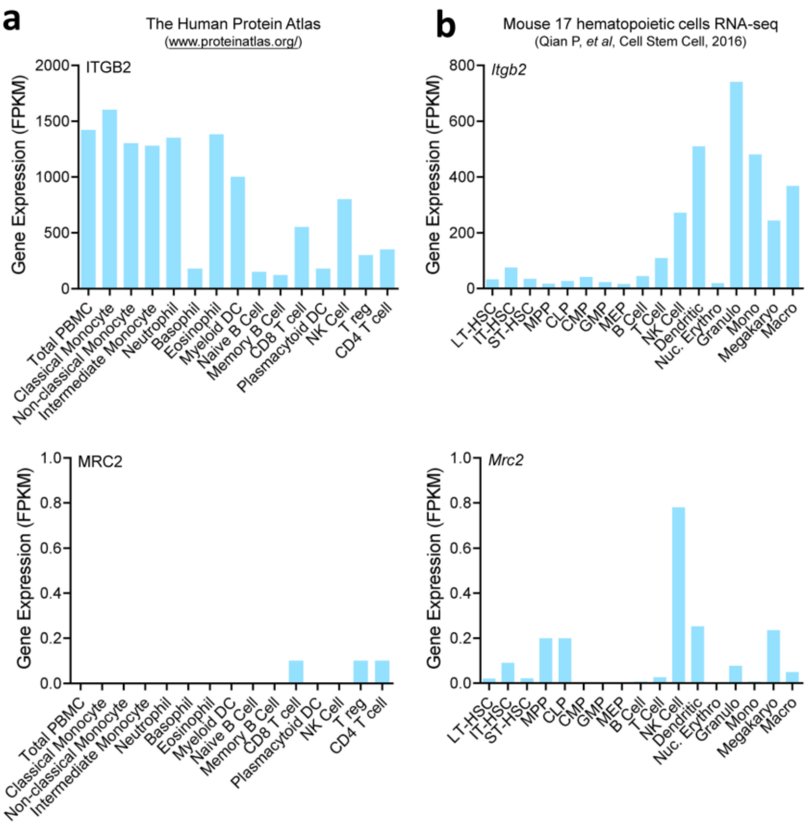

Supplementary Figure 21. Gene expression analyses of ITGB2 and MRC2 in normal hematopoietic cells. **a,** Expression of ITGB2 and MRC2 in human normal hematopoietic cells isolated for human protein atlas. **b,** Expression of ITGB2 and MRC2 in murine normal hematopoietic cells.

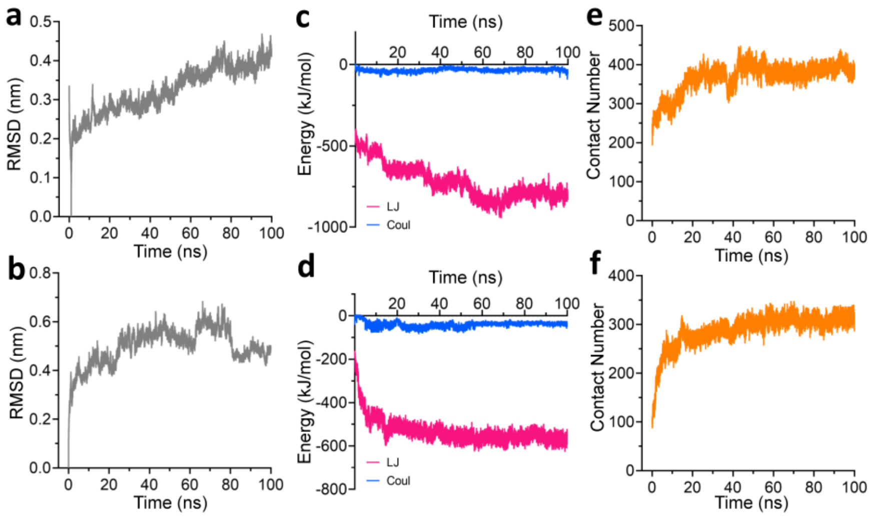

Supplementary Figure 22. Molecular dynamic and molecular docking simulation of GDYO nanosheets and ITGB2/MRC2. Root mean square deviation (RMSD, **a-b**), binding energy (**c-d**) and contact number (**e-f**) analysis in MD simulation of the interactions between GDYO nanosheets and ITGB2 (**a, c, e**) or MRC2 (**b, d, f**).

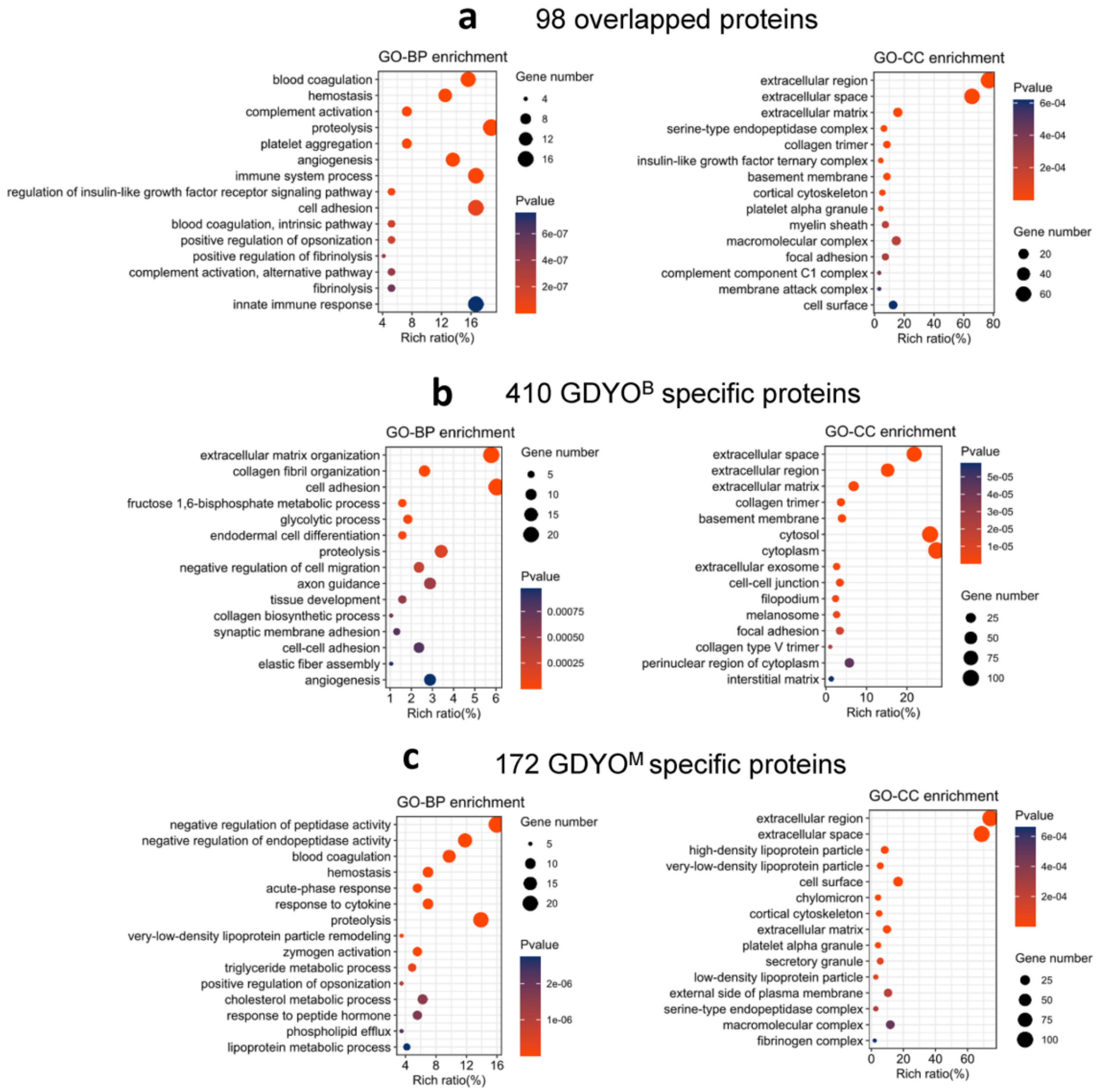

Supplementary Figure 23. GO analysis of GDYO-absorbed proteins in FBS or mouse plasma. **a**, Overlapped proteins of GDYO-absorbed proteins in FBS and mouse plasma. **b, c**, Specific proteins of GDYO-absorbed proteins in FBS **(b)** or mouse plasma **(b)**. The statistical tests were one-sided and adjustments were made for multiple comparisons.

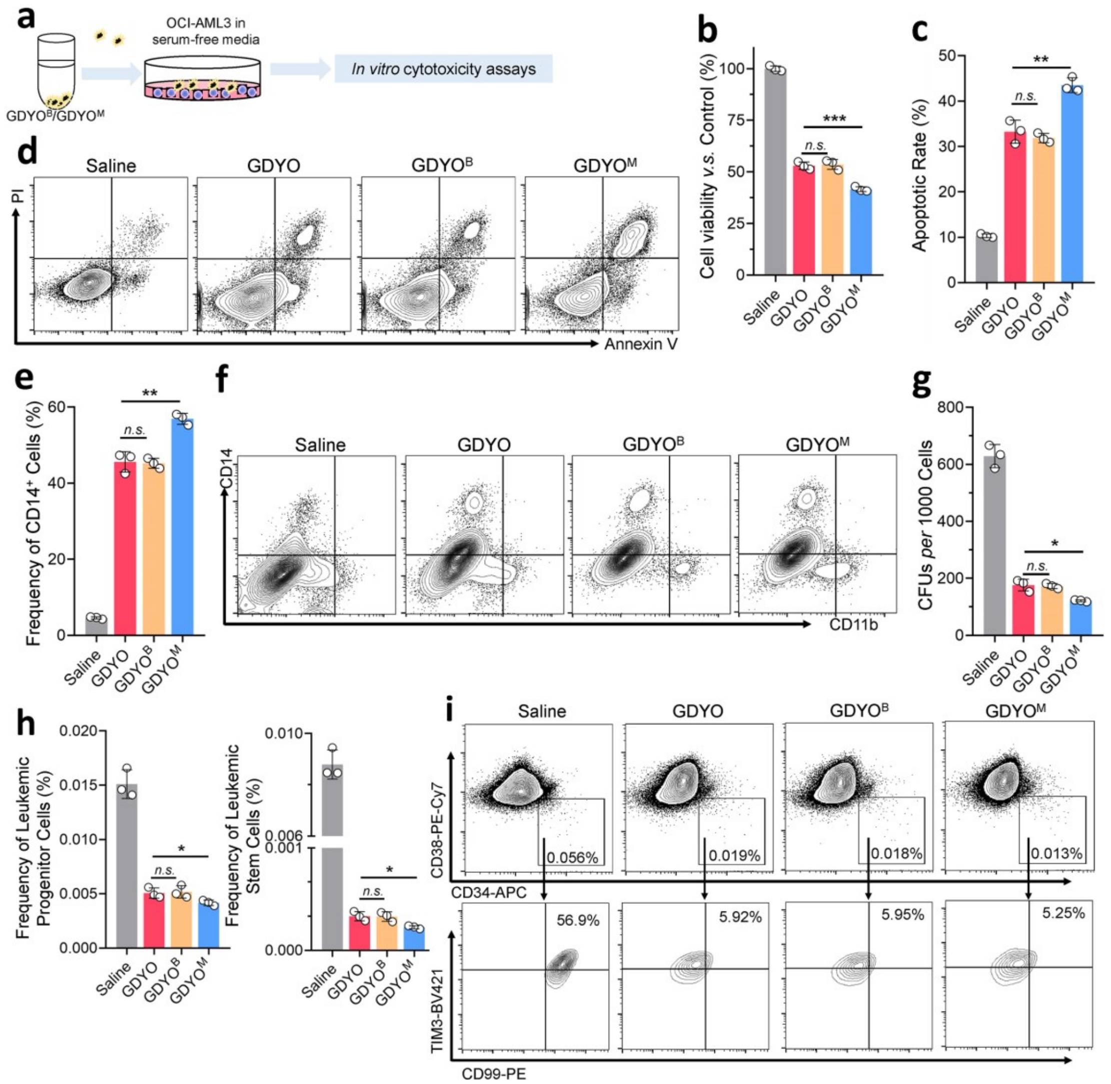

Supplementary Figure 24. *In vitro* cytotoxicity assays of GDYO/GDYO^{B}/GDYO^{M} against OCI-AML3 in serum-free media. a, Diagram to detect the *in vitro* cytotoxicity of GDYO^{B}/GDYO^{M} towards OCI-AML3 in serum-free media. **b.** Cell viability in OCI-AML3 treated with 20 μg/mL GDYO/GDYO^{B}/GDYO^{M} for 48 h. **c-d.** Apoptotic rates **(c)** and representative flow images **(d)** in OCI-AML3 treated with 20 μg/mL GDYO/GDYO^{B}/GDYO^{M} for 48 h. **e-f.** Frequency of CD14^{+} cells **(e)** and representative flow images **(f)** in OCI-AML3 treated with 20 μg/mL GDYO/GDYO^{B}/GDYO^{M} for 48 h. **g,** CFU colonies after 10 d culture in methylcellulose medium for OCI-AML3. **h-i.** Frequency **(h)** and representative flow images **(i)** of leukemic progenitor cells and leukemic stem cells in OCI-AML3 treated with 20 μg/mL GDYO/GDYO^{B}/GDYO^{M} for 72 h. *n* = 3 biologically independent experiments. The data were shown as the mean ± SD. Statistical significance was tested with a two-tailed, unpaired Student's *t* test. *n.s.* no significant, *$P < 0.05$, **$P < 0.01$, ***$P < 0.001$.

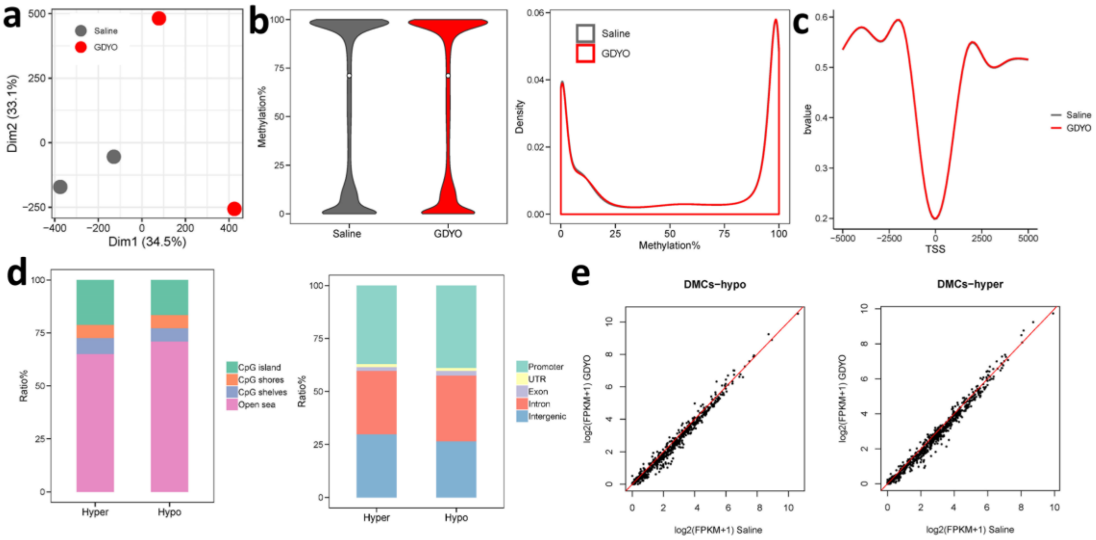

Supplementary Figure 25. Methylome analysis in GDYO-treated OCI-AML3 cells. a. PCA analysis for differentially methylated sites in saline or GDYO-treated OCI-AML3. **b.** Global methylation distribution in saline or GDYO-treated OCI-AML3 cells. **c-d.** Genomic distributions of differentially methylated sites. **e.** Correlation of hypomethylated and hypermethylated sites between saline and GDYO-treated OCI-AML3 cells.

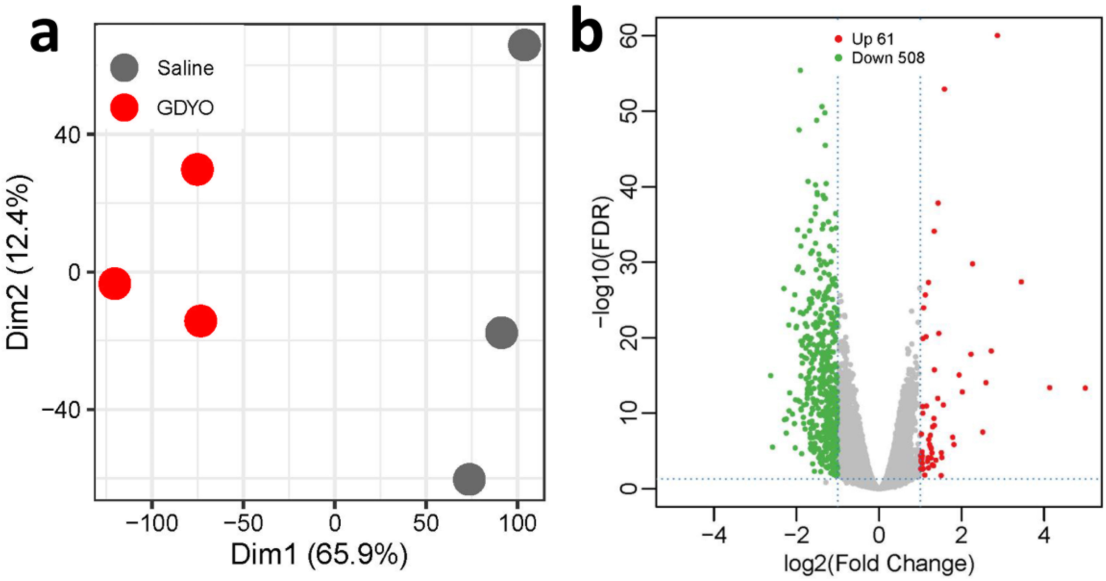

Supplementary Figure 26. Transcriptome analysis for GDYO-treated OCI-AML3. a. PCA analysis for RNA expression in saline or GDYO-treated OCI-AML3. **b.** Volcano plots of up- and down-regulated genes under GDYO treatment.

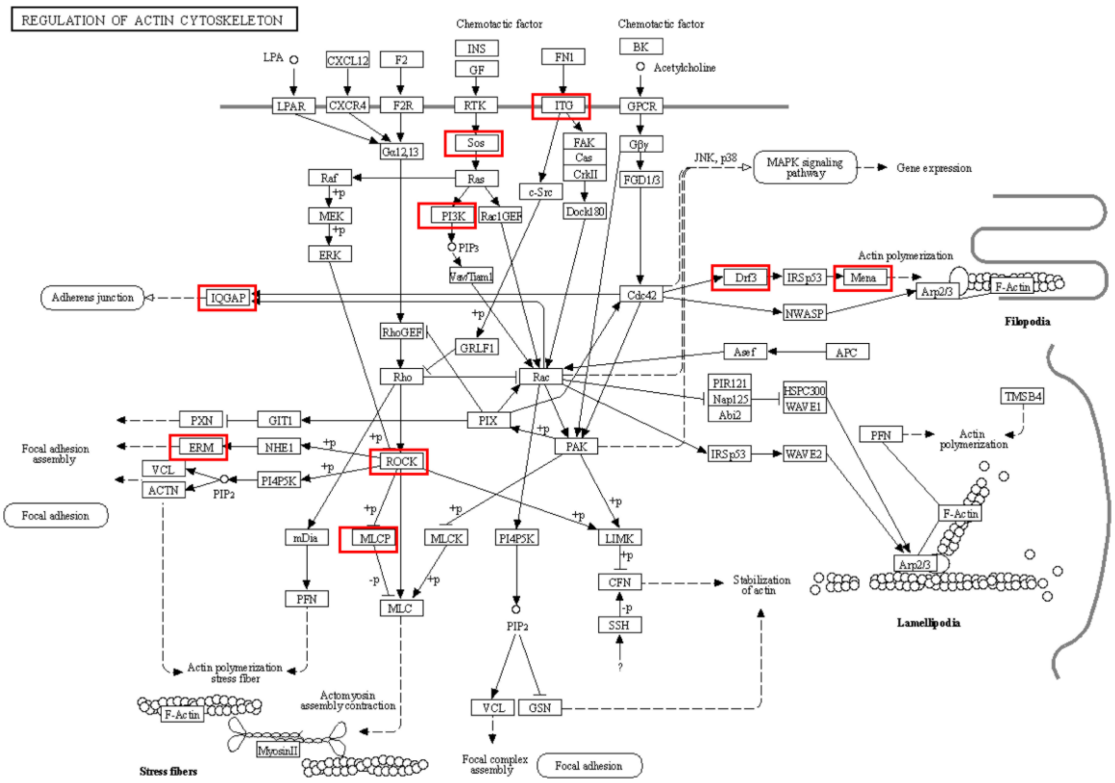

Supplementary Figure 27. Regulation of actin cytoskeleton pathway from KEGG analysis. The red boxes represent the down-regulated genes under GDYO treatment.

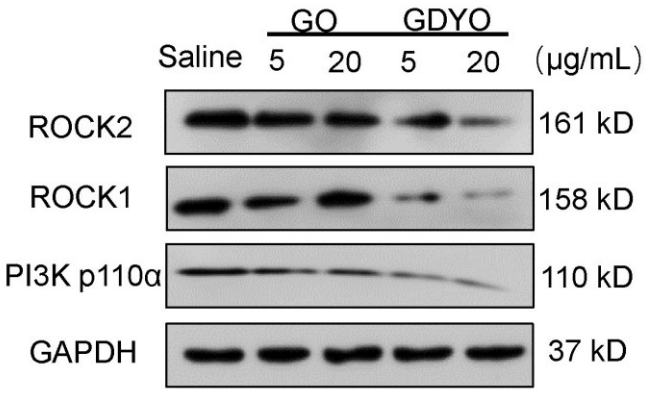

Supplementary Figure 28. Immunoblotting verifications of genes correlated with actin cytoskeleton organization in protein level.

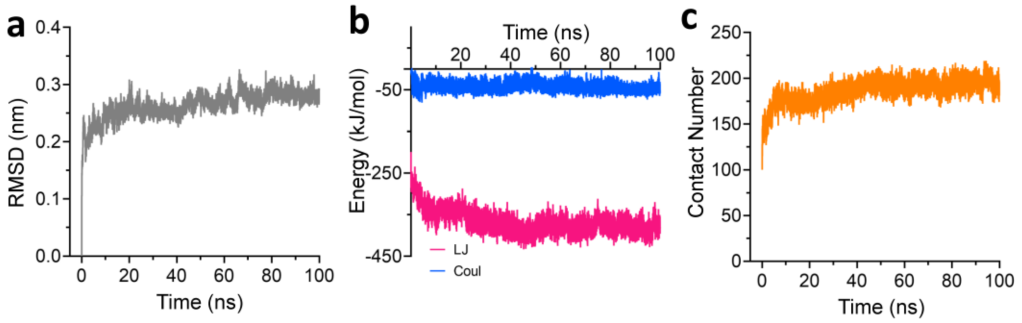

Supplementary Figure 29. Molecular dynamic and molecular docking simulation of GDYO/GO nanosheets and actin. Root mean square deviation (RMSD, **a**), binding energy (**b**) and contact number (**c**) analysis in MD simulation of the interactions between GDYO nanosheets and actin.

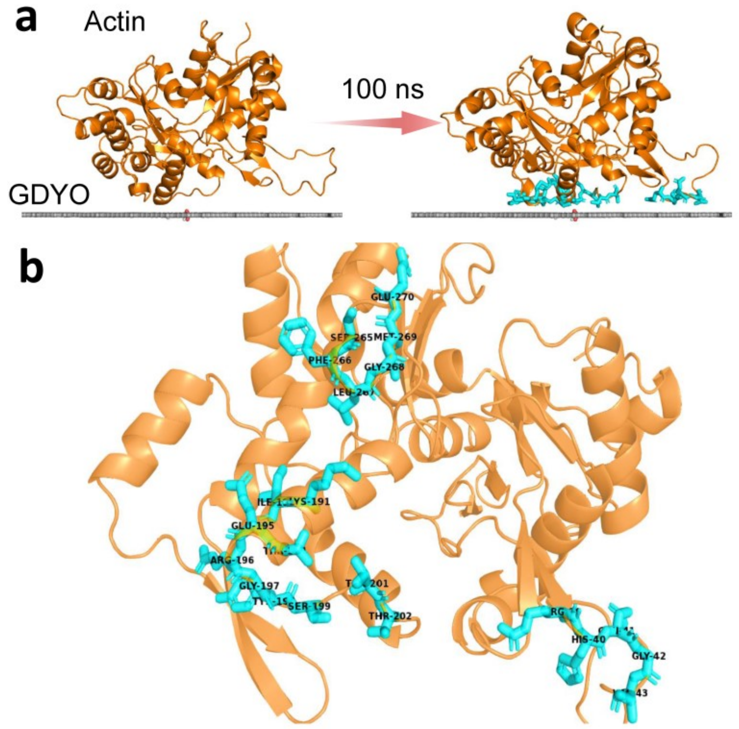

Supplementary Figure 30. Molecular dynamic (MD) simulation of GDYO nanosheet and actin. a. Side view of MD showing the adsorption of the actin protein onto GDYO nanosheet. **b.** Simulated binding site of GDYO onto actin monomer.

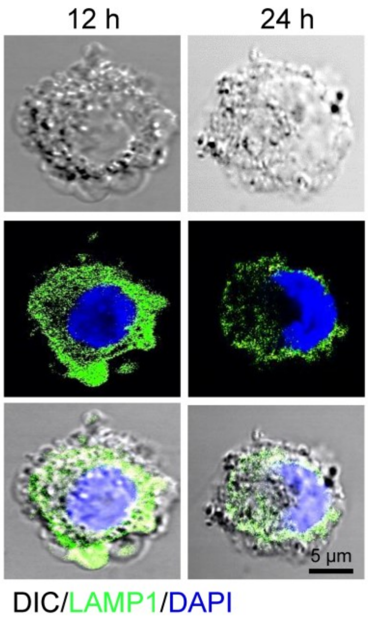

Supplementary Figure 31. Immunofluorescence confocal images with DIC component in OCI-AML3 treated by GDYO at 12 and 24 h. At least 10 images each group were taken. Scale bar, 5 μm.

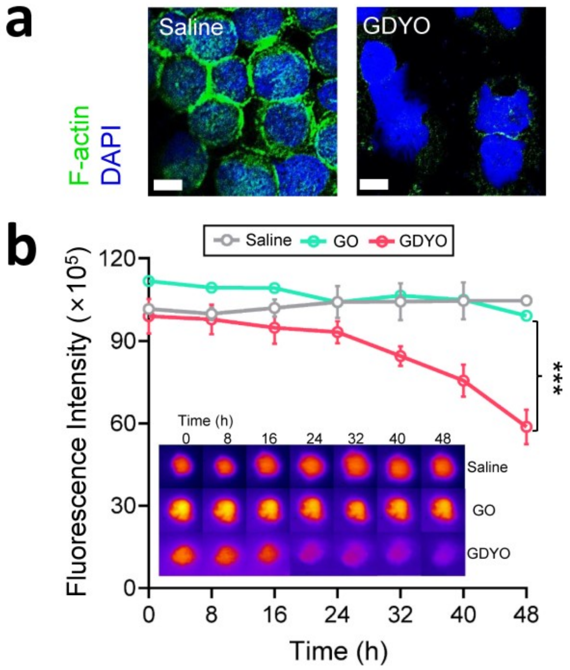

Supplementary Figure 32. GDYO nanosheets disrupted F-actin organization of *DNMT3A* mutant AML cells. a, Representative F-actin immunofluorescence confocal images of OCI-AML3 cells treated by GDYO for 48 h. Scale bar, 10 μm. **b.** Live imaging of F-actin in OCI-AML3 cells treated by GO or GDYO within 48 h. Scale bar, 10 μm. $n = 20$ biologically independent cells. The data were shown as the mean ± SD. Statistical significance was tested with a two-tailed, unpaired Student's t test. ***$P < 0.001$.

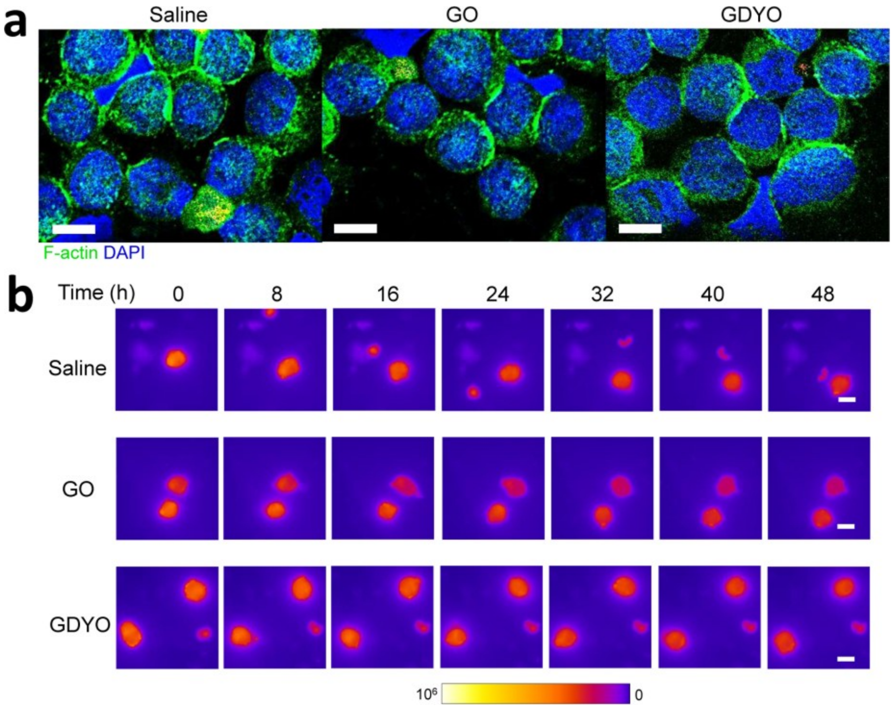

Supplementary Figure 33. GDYO did not disrupt F-actin organization in *DNMT3A*-wt AML cells. a, Representative F-actin immunofluorescence confocal images of HL-60 cells treated by GDYO for 48 h. At least 10 images each group were taken. Scale bar, 10 μm. **b.** Live imaging of F-actin in HL-60 cells treated by GO or GDYO within 48 h. At least 20 images each group were taken. Scale bar, 10 μm.

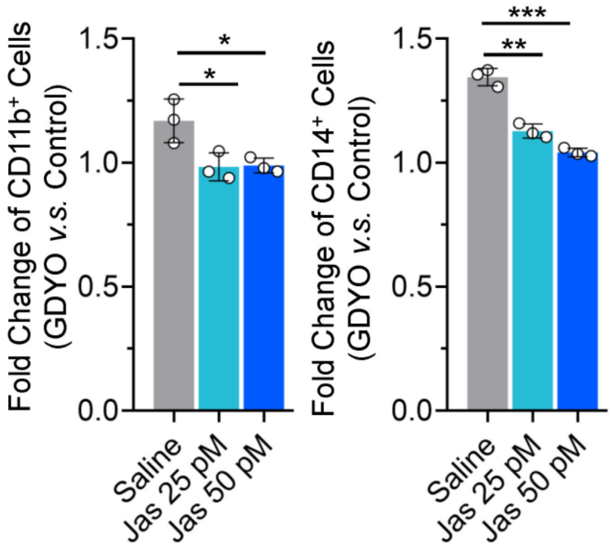

Supplementary Figure 34. Fold change of cell differentiation for GDYO *v.s.* control is rescued by Jas. $n = 3$ biologically independent experiments. The data were shown as the mean ± SD. Statistical significance was tested with a two-tailed, unpaired Student's t test. $^{*}P < 0.05$, $^{**}P < 0.01$, $^{***}P < 0.001$.

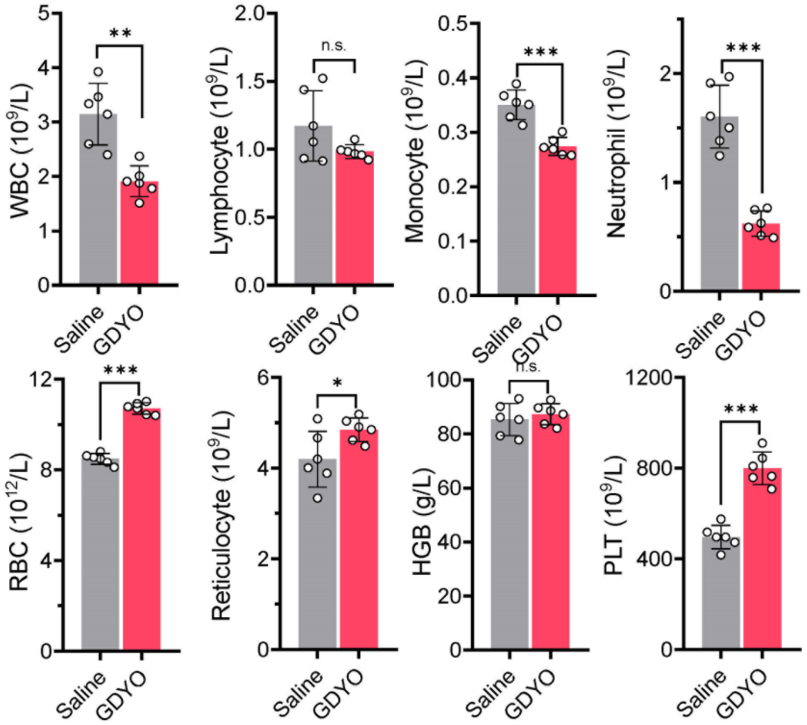

Supplementary Figure 35. Hematological data in AML mice. The AML NSG mice were treated with saline or GDYO. WBC, white blood cell, RBC, red blood cell, HGB, hemoglobin, PLT, platelets. $n = 6$ biologically independent animals. The data were shown as the mean ± SD. Statistical significance was tested with a two-tailed, unpaired Student's t test. *n.s.* no significant, $^*P < 0.05$, $^{**}P < 0.01$, $^{***}P < 0.001$.

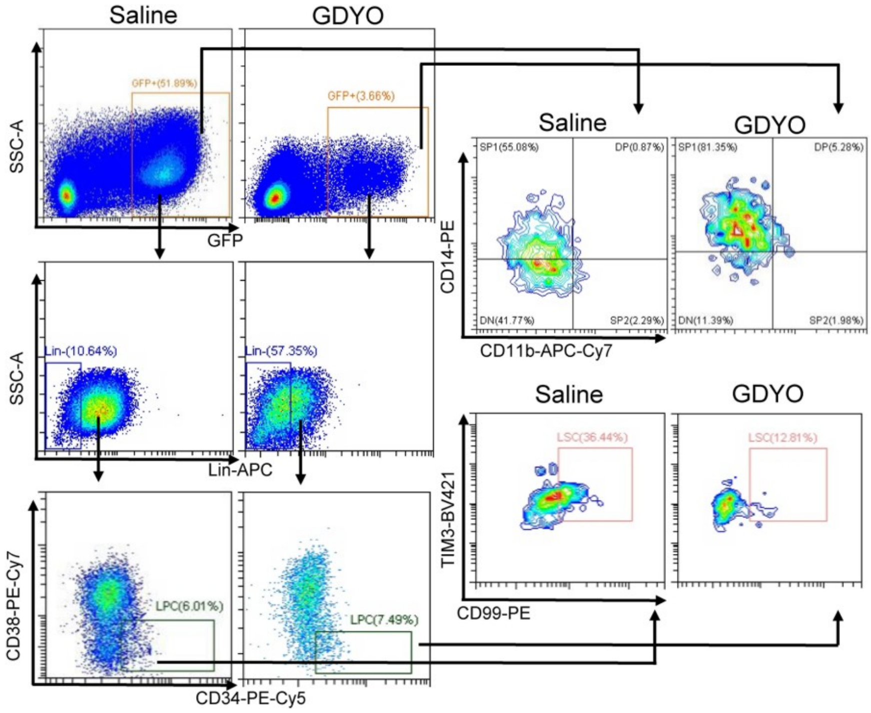

Supplementary Figure 36. Representative flow cytometry images of spleen total mononuclear cells isolated from AML mice treated by saline or GDYO.

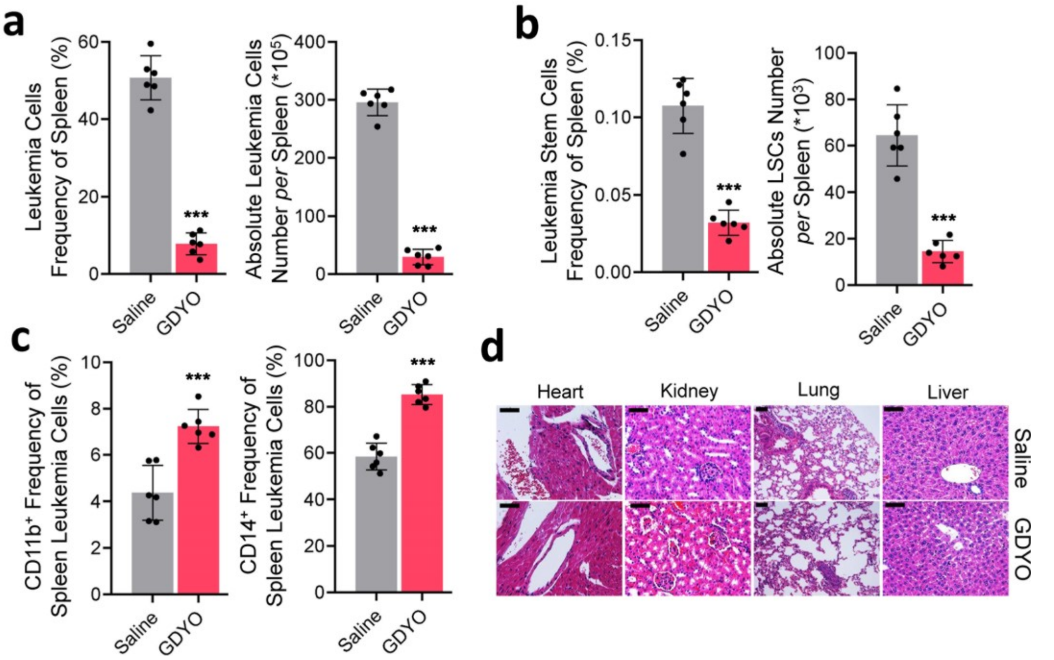

Supplementary Figure 37. Analysis of AML mice treated by saline or GDYO. a-c, Quantification of spleen leukemia cells **(a)**, leukemic stem cells **(b)** and myeloid differentiation **(c)**. **d,** H&E sections of main non-immune organs in mice with GDYO treatment. Scale bar, 10 μm. $n = 6$ biologically independent animals. The data were shown as the mean ± SD. Statistical significance was tested with a two-tailed, unpaired Student's t test. ***$P < 0.001$.

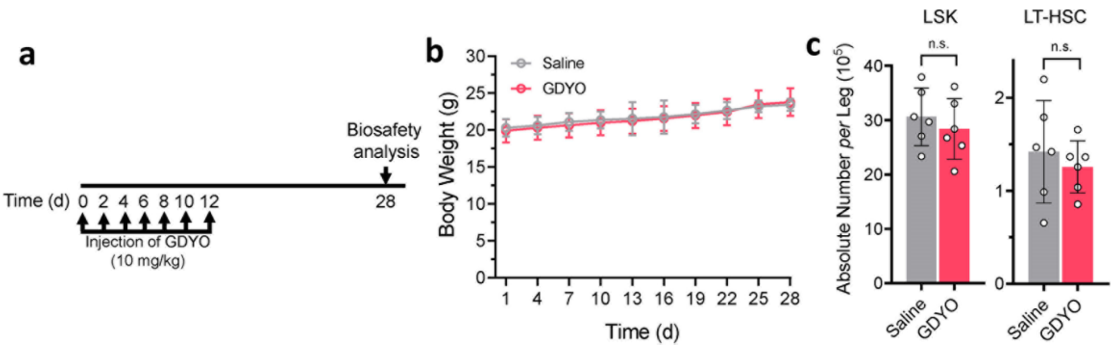

Supplementary Figure 38. Biosafety assessment of GDYO in mice. a, Schematic illustration of the *in vivo* experimental design. **b,** The body weights of C57BL/6 mice injected with saline or GDYO in 28 days. **c,** Absolute number of LT-HSCs (long-term HSCs) in mice injected with GDYO after sacrifice. $n = 6$ biologically independent animals. The data were shown as the mean ± SD. Statistical significance was tested with a two-tailed, unpaired Student's t test. *n.s.* no significant.

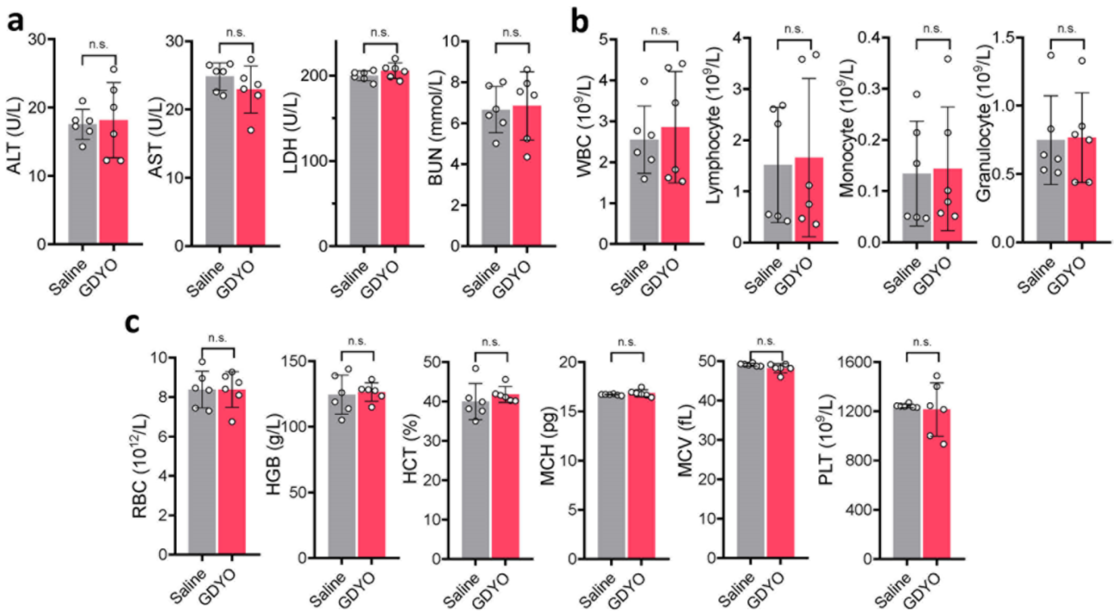

Supplementary Figure 39. Blood analysis of C57BL/6 mice injected with GDYO. a, Blood biochemical analysis of mice with GDYO treatment. ALT, alanine aminotransferase, AST, aspartate aminotransferase, LDH, lactate dehydrogenase, BUN, blood urea nitrogen. **b-c,** Hematological data of GDYO-treated mice. WBC, white blood cell, RBC, red blood cell, HGB, hemoglobin, HCT, hematocrit, MCH, mean corpuscular hemoglobin, MCV, mean corpuscular volume, PLT, platelets. $n = 6$ biologically independent animals *per* group. The data were shown as the mean ± SD. Statistical significance was tested with a two-tailed, unpaired Student's t test. *n.s.* no significant.

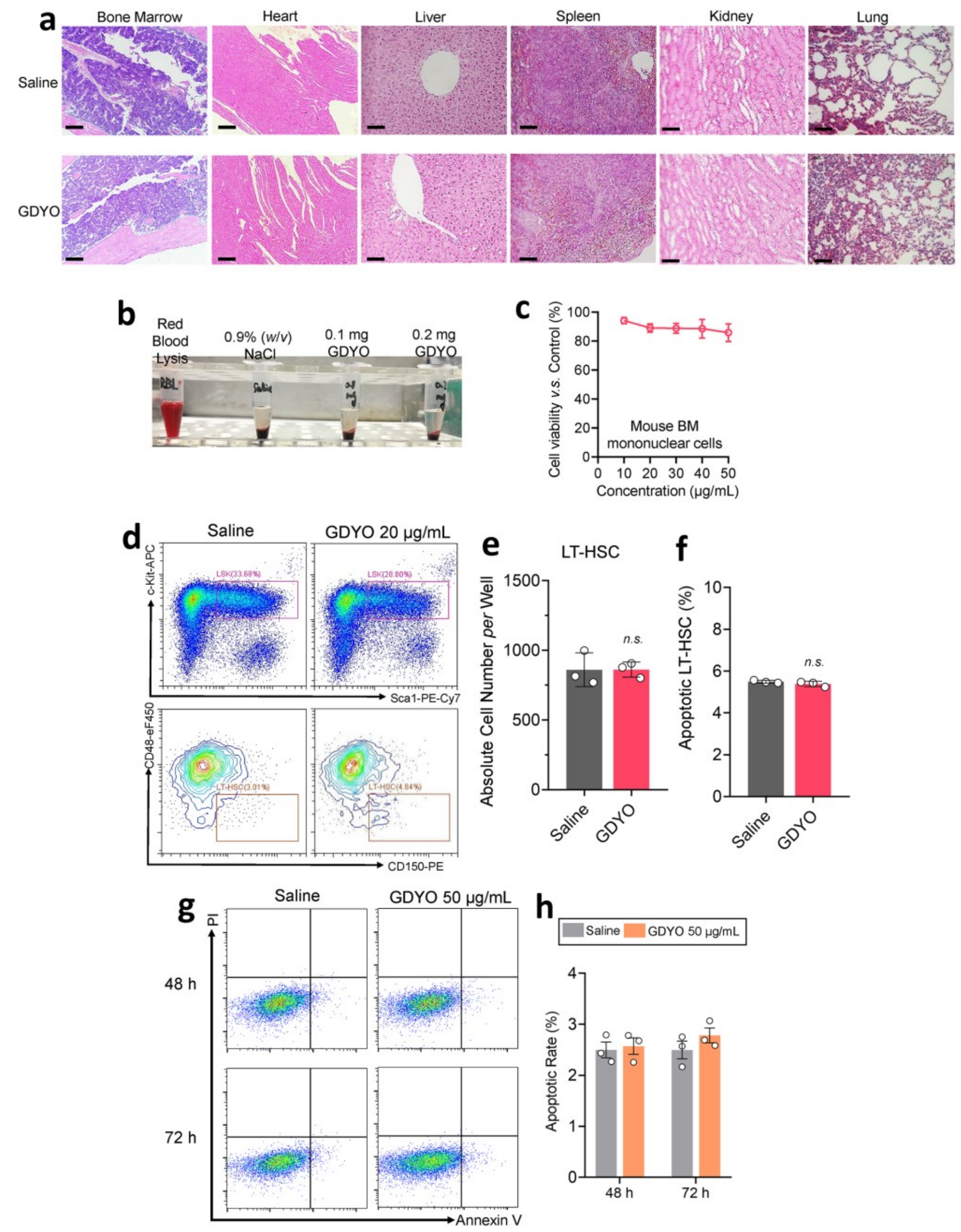

Supplementary Figure 40. Biosafety assessment of GDYO. a, H&E sections in main organs of mice with GDYO treatment. Scale bar, 10 μm. **b,** Images of hemolysis analysis of GDYO with whole blood for 8 h. **c,** Cell viability test in mouse BM and human PB mononuclear cells which were incubated with GDYO for 24 h. The data were shown as the mean ± SD. Statistical significance was tested with a two-tailed, unpaired Student's *t* test. *n* =3 biologically independent animals *per* group. **d,** Representative flow cytometry images of LSK and LT-HSC after GDYO co-incubation for 28 d. **e, f,** Absolute number **(e)** and apoptotic frequency **(f)** of LT-HSC after GDYO co-incubation for 28 d. **g, h,** Representative flow cytometry images **(g)** and apoptotic frequency **(h)** of human neuron cell line SH-S5Y5 after GDYO co-incubation for 48 and 72 h. *n* = 3 biologically independent experiments. The data were shown as the mean ± SD. Statistical significance was tested with a two-tailed, unpaired Student's *t* test. *n.s.* no significant.
