# Supplementary material for: Graphdiyne oxide nanosheets display selective anti-leukemia efficacy against DNMT3A-mutant AML cells
Source: Nat Commun. 2022 Sep 26;13:5657. doi: 10.1038/s41467-022-33410-w (PMC9512932; doi:10.1038/s41467-022-33410-w)
Supplement: Supplementary file 3 — Description of Additional Supplementary Files [file 41467_2022_33410_MOESM3_ESM.docx]

**Description of Additional Supplementary Files**

Supplementary Data 1: A list of proteins binding to GO or GDYO in OCI-AML3.

Supplementary Data 2: A list of proteins binding to GDYO in FBS or mouse plasma.

Supplementary Data 3: A list of differentially expressed genes in GDYO-treated OCI-AML3.

Supplementary Data 4: A list of gene functional annotations in GDYO-treated OCI-AML3.

Supplementary Data 5: A list of antibodies used in manuscript.

Supplementary Movie 1: Live imaging of PBS-treated OCI-AML3-LifeActin 48 h.

Supplementary Movie 2: Live imaging of GDYO-treated OCI-AML3-LifeActin 48 h.

Supplementary Movie 3: Live imaging of GO-treated OCI-AML3-LifeActin 48 h.

Supplementary Movie 4: Live imaging of PBS-treated HL-60-LifeActin 48 h.

Supplementary Movie 5: Live imaging of GDYO-treated HL-60-LifeActin 48 h.

Supplementary Movie 6: Live imaging of GO-treated HL-60-LifeActin 48 h.
